# Early Readmissions after Esophagectomy for Esophageal Adenocarcinoma: Does Facility Case-Volume Matter?

**DOI:** 10.1155/2020/8072682

**Published:** 2020-01-29

**Authors:** Kwabena Oware Adu-Gyamfi, Chaitanya Pant, Abhishek Deshpande, Hassanain Jassim, Mojtaba Olyaee

**Affiliations:** ^1^Department of Internal Medicine, HSHS St. Mary's Hospital Medical Center, Green Bay, WI, USA; ^2^Division of Gastroenterology, Hepatology and Motility, Department of Internal Medicine, University of Kansas Medical Center, Kansas City, KS, USA; ^3^Department of Internal Medicine, Cleveland Clinic, Cleveland, OH, USA; ^4^Department of Surgery, HSHS St. Vincent Hospital, Green Bay, WI, USA

## Abstract

Increased esophagectomy procedures over the past four decades have correlated with the rise in incidence of esophageal adenocarcinoma. Despite advances in technology and procedural expertise, esophagectomy remains a high-risk surgical procedure. Higher volume facilities have more experience with esophagectomy and would be expected to have a lower incidence of surgical complications and attendant morbidity and mortality. By analyzing information from a nationwide United States hospital database, we sought to find out if there is a significant difference between facilities stratified by case volume, with regards to 30-day readmission after esophagectomy. The findings of this study indicated that even with a large applied differential, early readmissions did not differ significantly between high- and low-volume centers. Also, analyzed and discussed were any associated demographic and comorbidity factors as they relate to early readmissions after esophagectomy for esophageal adenocarcinoma across the country. This is the first study to specifically address these variables.

## 1. Introduction

The incidence of esophageal adenocarcinoma in the United States has increased dramatically since the 1970s and has been attributed to a contemporaneous rise in the prevalence of obesity, Barrett's esophagus and gastroesophageal reflux disease [1]. Concurrently, there has been an increased number of esophagectomies performed for esophageal cancer. An analysis of United States hospital data from 2001 to 2010 demonstrated an overall increased trend in esophagectomy procedures performed for this indication with a concomitant reduction in the mortality rate [2].

Esophagectomy for esophageal cancer remains a high-risk procedure with a substantial morbidity and mortality rate [3]. Previous studies including meta-analysis suggest that esophagectomy performed at low-volume hospitals was associated with a significant increase in incidence of in-hospital and 30-day mortality [4]. More recently, with hospital case volume assigned to a threshold of 10 cases per year, there was no significant difference noted in risk-adjusted outcomes between high-volume centers (HVCs) and low-volume centers (LVCs) [2].

Hospital readmissions following esophagectomy range from 12-25% and are associated with decreased patient survival [3, 5, 6]. However, data regarding risk factors for early (30-day) readmission after esophagectomy have been conflicting. Also, it is not known whether the incidence of early readmission following esophagectomy is related to the annual case volume of the performing center.

We utilized a nationwide readmission database to study 30-day readmissions following esophagectomy performed for esophageal cancer. The aim of this study was two-fold; first, to compare the incidence of 30-day readmissions among centers stratified by annual case volume. Second, to study patient demographics, comorbidities, and associated diagnosis and procedure codes as they relate to early readmissions following an esophagectomy.

## 2. Methods

### 2.1. Data Source

We obtained data for this study from the 2013 Nationwide Readmissions Database (NRD), Healthcare Cost and Utilization Project (HCUP), Agency for Healthcare Research and Quality (AHRQ), which is designed to provide information on national readmission rates in the U.S. The NRD is an annual database and contains data from community, specialty, and public hospitals and academic medical centers including pediatric hospitals. The NRD is a “discharge-level” file, meaning that each record represents a single discharge corresponding to an inpatient stay. Should the patient have multiple hospital visits in a particular year, the NRD will include separate records corresponding to each inpatient stay. The NRD includes discharges on patients aged 1 year and older. Each discharge entry includes patient demographic details, 1 primary discharge diagnosis (based on the International Classification of Diseases, Ninth Revision, and Clinical Modification, ICD-9-CM, diagnosis codes), 1 to 24 ICD-9-CM secondary diagnoses, 1 to 15 ICD-9-CM procedural codes, hospitalization information, and hospital data. Discharge weights are provided to generate national estimates; it estimates roughly 36 million discharges in the United States. All data in this study are reported as national level estimates. The study excluded any protected health information and was deemed exempt from institutional board review.

### 2.2. Esophagectomy Definition, Study Demographics, and Readmissions

For the purpose of this study, we included all patients 18 years of age and older who underwent esophagectomy, total or partial, performed for the indication of esophageal cancer during the period January 1–December 31, 2013 and were discharged alive following surgery. We used previously described International Classification of Diseases, Ninth Revision, Clinical Modification (ICD-9-CM) diagnostic and procedural codes [2]. These included ICD-9-CM diagnosis codes for esophageal cancer 150.0–150.5, 150.8, and 150.9 and ICD-9-CM procedure codes for partial esophagectomy 42.41, 42.5, 42.51, and 42.52 and for total esophagectomy 42.42 and 42.11.

The total number of esophageal resections was calculated for each hospital. Hospitals were then classified as either HVCs (10 or more cases during the study period) or LVCs (fewer than 10 cases during study period). For each esophagectomy, it was determined if the patient had a subsequent readmission in a 30-day period.

Patient demographics, diagnoses, procedures, and hospitalization data were obtained for the extracted cases. The occurrence of postoperative complications (POC) following esophagectomy [2], the incidence of patient comorbidities using the Elixhauser scale [7], and the requirement for intensive care unit (ICU) stay [8] were assessed for using previously described methods.

We calculated hospital length of stay (LOS) and hospitalization costs (obtained by multiplying total charges by cost-to-charge ratios and rounded to the nearest $1,000). We also determined the most frequently associated diagnostic and procedural codes associated with early readmissions. Specifically, in patients with a 30-day readmission after esophagectomy, we extracted all ICD-9-CM primary diagnoses codes that occurred with a frequency of 2% or greater. Similarly, we also extracted all ICD-9-CM procedural codes that occurred at a frequency of 3% or greater.

### 2.3. Statistical Analysis

Statistical analyses were performed using SAS version 9.2 (SAS Institute, Cary, North Carolina, USA). The Kolmogorov–Smirnov test was used to determine the normality of the data elements under study. The Kruskal–Wallis and chi-square test were used to compare differences in continuous and categorical variables, respectively. Univariate and multivariate logistic regression analyses were performed with the presence or absence of 30-day readmission as the dichotomous outcome variable. Covariates that were tested for association with readmission included variables that were identified as significant at the univariate level. We tested all between-variable estimated correlation coefficients and determined that multicollinearity was not a problem. Odds ratios (ORs), adjusted odds ratios (aORs), and 95% confidence intervals (CIs) were reported to identify the strength and significance of readmission and other covariates on the likelihood of an association. The threshold for significance for all analyses was *p* < 0.01.

## 3. Results

In 2013, there were a total 2,017 esophagectomies performed for indication of esophageal cancer meeting our study criteria. Three hundred and forty-seven patients were readmitted within 30 days of discharge. Comparing HVC versus LVC, the incidence of 30-day readmissions following esophagectomy did not differ significantly (16.9% vs. 17.4%; *p*=0.77). HVC however had a lower incidence of POC (43.80% vs. 58.30%; *p* < 0.001) and a shorter average length of stay (9 days vs. 11 days; *p* < 0.001) compared with LVC. Infectious etiologies were the leading cause for readmissions. Comparing patient demographics from HVC versus LVC, no significant difference was found in median age (65 years, (interquartile range; IQR 13) vs. 64 (13) years; *p*=0.08), median comorbidity score (2 (2) vs. 2 (3); *p*=0.41), median time to readmission (8 (10) days vs. 9 (13) days; *p*=0.02), or median hospital costs ($40,000 ($23,000) vs. $40,000 ($31,000); *p*=0.54). The findings mentioned above are summarized in Tables 1 and 2.

Table 3 lists the most frequent procedural codes that were associated with 30-day readmissions following an esophagectomy.

Comparing patients who did not undergo a 30-day readmission following esophagectomy to those who did, the major demographic characteristics, illness severity, and hospital course are outlined in Table 4.

For patients undergoing a 30-day readmission, the major associated comorbidities and postoperative complications are outlined in Table 5.

## 4. Discussion

Readmission after surgery continues to be a significant healthcare problem, and recent policy changes that include substantial financial penalties have made readmission in a crucial pay-for-performance program in the U.S [9]. Our results indicate that the incidence of 30-day readmission after an esophagectomy continues to be high at 17.20% and is consistent with previous finds [5, 6]. The novel aspect of this study is that it compares the incidence of early readmissions following esophagectomy among centers stratified by annual case volume. Patient characteristics at both the centers did not differ significantly in regard to median age, comorbidity score, and the median time to readmission or costs. Importantly, the incidence of 30-day readmissions did not significantly differ between the two types of centers. Even when we reassigned the case threshold for HVCs to ≥30 cases per year, the difference did not achieve statistical significance (data not shown). While HVCs did demonstrate an advantage in shorter hospital stays and a lower incidence of POCs, these have been previously shown to be nonsignificant in multivariate analysis [2]. While the definition of high- and low-volume centers in the literature is extremely variable, these data question the perceived benefits of esophagectomy performed at a HVC.

We analyzed the diagnoses and procedures associated with 30-day readmission following an esophagectomy. An infectious etiology was noted as the primary cause for readmission in 17.5% of cases including septicemia (6.1%), pneumonia (5.1%), and empyema (2.6%). Postoperative pulmonary complications are recognized as the most frequent systemic complications after esophagectomy, and intensive preoperative respiratory rehabilitation has been demonstrated to decrease these complications [10]. Esophagogastroduodenoscopy (EGD) was noted to be the most frequently performed procedure in 19.3% of patients following a 30-day readmission after esophagectomy. Following an esophagectomy, up to 10% of patients developed an anastomotic leak and up to 22% developed an anastomotic stricture; 34% of these strictures are manifested within 1–3 months [11]. In this context, we noted that 6.3% of our readmitted cohort underwent esophageal stenting and 3.4% underwent esophageal dilation.

The overall healthcare burden associated with 30-day readmissions after esophagectomy was substantial. This was realized in the significantly high hospital-related stays and costs as well as unfavorable outcomes including ICU stay and death. Mortality and ICU stay were strongly associated with an infectious, cardiopulmonary, or renal complication. Factors associated with early readmission included lengthier hospital stays and higher hospital costs following the index surgery as well as a greater incidence of comorbidities and POCs. All of these likely represent a more complicated initial hospital course and consequently portend early readmission. Therefore, the subset of patients displaying these high-risk characteristics would possibly benefit from more detailed evaluation before discharge.

On multivariate analysis, we observed that cardiovascular and renal complications present at the time of esophagectomy were independently associated a higher incidence of subsequent 30-day readmission. A higher preoperative comorbidity score has been inconsistently reported as a risk factor for readmission. For example, Fernandez et al. reported that a Charlson comorbidity index of 3+ was strongly associated with readmission [5]. However, a subsequent study reported that the only significant preoperative predictor of readmission was delivery of induction therapy [6]. Similarly, the incidence of POCs has been reported to be significantly associated with an increased risk for 30-day readmission [12]. However, we were unable to find evidence for this in multivariate analyses.

There are limitations to this study; several of these have previously been elaborated upon in an analysis of the related Nationwide Inpatient Sample (NIS) database [2]. The HCUP family of databases including the NIS and NRD do not contain data pertaining to tumor type and stage, pulmonary function, performance status, or the use of neoadjuvant therapy. The study relies solely on the use of ICD-9-CM codes for case and procedure identification. There is a lack of information on the surgeons' specialty or case volume, which may be more closely related to outcomes than hospital volume [13]. Specific to the NRD is a lack of information on patient race, the limitation of data to a single calendar year, and the possibility that patients were readmitted to a hospital in a different state, whereupon they would lose their initial identification code in the database. However, the NRD does have an advantage over the frequently queried Surveillance, Epidemiology, and End Results (SEER)-Medicare dataset, which excludes patients <65 years of age [6].

## 5. Conclusions

Readmissions within a 30-day period are common after esophagectomy performed for esophageal cancer. Even with a large applied differential, early readmissions did not differ significantly between high- and low-volume centers. Patients with a complicated medical course after esophagectomy, greater comorbidities, and POCs demonstrate a higher incidence of readmission; cardiovascular and renal comorbidities are independently associated with an increased risk. Infectious etiologies are the leading cause of early readmission, while EGD with stent placement and dilation is the most frequently performed medical procedure. Based on patient characteristics and outcomes during initial hospital stay, patients at high risk for readmission may be identified and should be evaluated carefully before clearing for hospital discharge.

## Figures and Tables

**Table 1:** Comparison of esophagectomy performed at high- and low-volume centers for esophageal cancer in patients 18 years of age and older who survived their initial hospital stay. High-volume centers were defined as performing 10 or more cases per year, while low-volume centers performed 9 or less cases annually. IQR interquartile range.

Variable	High‐volume center	Low‐volume center	*p* value
Esophagectomy (annual case volume)	898	1119	
Cases without readmission	746 (83.1%)	924 (82.6%)	0.77
Cases with 30-day readmission	152 (16.9%)	195 (17.4%)	0.77
Median patient age in years (IQR)	65 (13)	64 (13)	0.08
Median time to readmission (IQR)	8 (10)	9 (13)	0.02
Median length of stay in days (IQR)	9 (5)	11 (8)	<0.001
Median costs (IQR)	$40,000 ($23,000)	$40,000 ($31,000)	0.54
Median comorbidity score (IQR)	2 (2)	2 (3)	0.41
Incidence of postoperative complications	43.80%	58.30%	<0.001

**Table 2:** Most frequent causes of 30-day readmission following esophagectomy as assessed by the primary ICD-9-CM diagnosis codes. ICD-9-CM international classification of diseases, ninth revision, and clinical modification.

ICD-9-CM code	Description	Frequency (%)
99749	Other digestive system complications	8.10
0389	Septicemia	3.80
99859	Postop infection	3.70
5609	Unspecified intestinal obstruction	3.30
486	Pneumonia	3.10
33812	Acute postthoracotomy pain	3.00
5109	Empyema without fistula	2.60
03849	Septicemia due to other Gram-negative organisms	2.30
56962	Mechanical complication of ostomy	2.20
27651	Dehydration	2.00
99739	Aspiration pneumonia	2.00

**Table 3:** Most frequent procedures performed in patients readmitted in a 30-day period following esophagectomy as assessed by the ICD-9-CM procedure codes.

ICD-9-CM code	Description	Frequency (%)
4513	Esophagogastroduodenoscopy	19.30
3404	Insertion of intercostal catheter for drainage	16.70
966	Total parenteral nutrition	16.30
9904	Packed red blood cell transfusion	12.40
9604	Insertion of endotracheal tube	11.10
9672	Continuous invasive mechanical ventilation for 96 consecutive hours or more	10.20
3897	Central venous catheter placement with guidance	8.60
3324	Endoscopic biopsy of bronchus	7.90
3491	Thoracentesis	7.20
4281	Insertion of permanent tube into esophagus	6.30
9671	Continuous invasive mechanical ventilation for 96 consecutive hours or less	5.40
8872	Echocardiogram	4.70
3322	Fiber-optic bronchoscopy	4.20
9915	Parenteral infusion	4.20
9703	Replacement of tube or enterostomy device of small intestine	3.80
3893	Venous catheterization	3.70
4422	Endoscopic dilation of pylorus	3.60
4292	Esophageal dilation	3.40
3323	Other bronchoscopy	3.20

**Table 4:** Comparison of patient characteristics and outcomes during initial hospitalization for esophagectomy and classified as either with or without a subsequent 30-day readmission.

Variable	Patients without readmission	Patients with readmission	*p* value
Median age in years (IQR)	64 (13)	65 (13)	0.1
Median LOS (IQR)	9 (6)	11 (9)	<0.001
Median costs (IQR)	$38,000 ($25,000)	$44,000 ($36,000)	<0.001
Median comorbidity score (IQR)	2 (3)	3 (2)	<0.001
Incidence of postoperative complications	49.70%	61.70%	<0.001
Requirement for ICU care	13.20%	21.00%	<0.001

IQR: interquartile range, ICU: intensive care unit, LOS: length of stay.

**Table 5:** Incidence of comorbidities and postoperative complications in patients following esophagectomy who were readmitted within a 30-day period compared with patients who did not undergo early readmission.

Comorbidity	Frequency; *p* value	Unadjusted OR (95% CI)
Diabetes mellitus with complications	4.3% vs. 1.5%; *p*=0.001	2.97 (1.55–5.70)
Congestive heart failure	11.6% vs. 4.3%; *p* < 0.001	2.94 (1.96–4.42)
Cardiac valvular disease	5.2% vs. 2.0%; *p*=0.001	2.71 (1.51–4.88)
Renal disease including renal failure	10.1% vs. 4.4%; *p* < 0.001	2.45 (1.61–3.74)
Peripheral vascular disease	7.8% vs. 4.3%; *p*=0.01	1.90 (1.20–3.01)
Postoperative complication		
Deep vein thrombosis	4.0% vs. 1.3%; *p*=0.001	3.15 (1.59–6.22)
Wound dehiscence	13.3% vs. 6.9%; *p* < 0.001	2.05 (1.42–2.94)
Anastomotic leak/mediastinitis	15.9% vs. 9.0%; *p* < 0.001	1.89 (1.36–2.64)
Dysphagia	20.5% vs. 12.3%; *p* < 0.001	1.83 (1.36–2.46)

OR: odds ratio, CI: confidence intervals.

## Data Availability

The data used to support the findings of this study are available from 2013 Nationwide Readmissions Database (NRD) of the Healthcare Cost and Utilization Project (HCUP), through the United States Agency for Healthcare Research and Quality (AHRQ).

## References

[1] Thrift A. P. (2016). The epidemic of oesophageal carcinoma: where are we now?. *Cancer Epidemiology*.

[2] Jafari M. D., Halabi W. J., Smith B. R. (2013). A decade analysis of trends and outcomes of partial versus total esophagectomy in the United States. *Annals of Surgery*.

[3] Finks J. F., Osborne N. H., Birkmeyer J. D. (2011). Trends in hospital volume and operative mortality for high-risk surgery. *New England Journal of Medicine*.

[4] Markar S. R., Karthikesalingam A., Thrumurthy S., Low D. E. (2012). Volume-outcome relationship in surgery for esophageal malignancy: systematic review and meta-analysis 2000–2011. *Journal of Gastrointestinal Surgery*.

[5] Fernandez F. G., Khullar O., Force S. D. (2015). Hospital readmission is associated with poor survival after esophagectomy for esophageal cancer. *The Annals of Thoracic Surgery*.

[6] Hu Y., McMurry T. L., Stukenborg G. J., Kozower B. D. (2015). Readmission predicts 90-day mortality after esophagectomy: analysis of surveillance, epidemiology, and end results registry linked to medicare outcomes. *The Journal of Thoracic and Cardiovascular Surgery*.

[7] Elixhauser A., Steiner C., Harris D. R., Coffey R. M. (1998). Comorbidity measures for use with administrative data. *Medical Care*.

[8] Kassam Z., Cribb Fabersunne C., Smith M. B. (2016). *Clostridium difficile* associated risk of death score (CARDS): a novel severity score to predict mortality among hospitalised patients with C. difficile infection. *Alimentary Pharmacology & Therapeutics*.

[9] Lucas D. J., Pawlik T. M. (2014). Readmission after surgery. *Advances in Surgery*.

[10] Inoue J., Ono R., Makiura D. (2013). Prevention of postoperative pulmonary complications through intensive preoperative respiratory rehabilitation in patients with esophageal cancer. *Diseases of the Esophagus*.

[11] Briel J. W., Tamhankar A. P., Hagen J. A. (2004). Prevalence and risk factors for ischemia, leak, and stricture of esophageal anastomosis: gastric pull-up versus colon interposition. *Journal of the American College of Surgeons*.

[12] Shah S. P., Xu T., Hooker C. M. (2015). Why are patients being readmitted after surgery for esophageal cancer?. *The Journal of Thoracic and Cardiovascular Surgery*.

[13] Rodgers M., Jobe B. A., O’Rourke R. W., Sheppard B., Diggs B., Hunter J. G. (2007). Case volume as a predictor of inpatient mortality after esophagectomy. *Archives of Surgery*.

